# Cyanoacrylate Associated Foreign Body Granulomatous Gastritis: A Report of Three Cases

**DOI:** 10.1155/2017/2753487

**Published:** 2017-01-19

**Authors:** Gunes Guner, Olcay Kurtulan, Taylan Kav, Cenk Sokmensuer, Gokhan Gedikoglu, Aytekin Akyol

**Affiliations:** ^1^Department of Pathology, Faculty of Medicine, Hacettepe University, Sıhhiye, 06100 Ankara, Turkey; ^2^Gastroenterology Division, Department of Internal Medicine, Faculty of Medicine, Hacettepe University, Sıhhiye, 06100 Ankara, Turkey

## Abstract

Granulomas are rarely seen in gastric biopsies mostly as an involvement of granulomatous diseases like sarcoidosis, Crohn's disease, infections, neoplasms, and vasculitis. Here, we claim cyanoacrylate as a foreign body type granuloma-causing agent in the stomach after vascular embolisation. We present cyanoacrylate associated gastric changes of three cases: two endoscopic biopsies and one gastric resection. In two cases, cyanoacrylate associated ulcers and granulomatous inflammation were observed in gastric mucosal biopsies following endoscopic examination after 7 months and 6 years of the glue injections, respectively. In the third case, the cyanoacrylate injection was performed 2 months prior to the surgery. Then the patient underwent distal pancreatectomy for pancreatic adenocarcinoma and during the operation a gastric mass was resected with a suspicion of tumoral infiltration. These three cases demonstrated that glue exposure causes active chronic inflammation with foreign body type granulomas, mucosal ulceration, and bleeding in the gastric mucosa. Even further, it can induce mass formation in the injection sites.

## 1. Introduction

Granulomas in the stomach can be due to several diseases, such as Crohn's disease, sarcoidosis, infections, lymphoma, adenocarcinoma, Whipple's disease, Langerhans cell histiocytosis, gastric perforation, and vasculitis [1]. Foreign bodies getting stuck in the gastrointestinal system are also well documented [2]. We hereby report another, possibly overlooked cause for granulomas in the stomach: cyanoacrylate injections.

Cyanoacrylate derivatives have been used as hemostatic or anastomotic agents [3]. In the clinical setting, typically very small amounts (0.3–0.5 mL) of cyanoacrylate, alone or in combination with alcohol and beta-blockers, are administered into the bleeding or varicose vessel to obstruct the lumen. The main complications include perforation, sepsis, erosions or ulcerations, pneumatosis, and stricture formation with dysphagia [4]. The morphological characteristics of cyanoacrylate injected vascular lesions (hemangiomas, vascular malformations) at different timepoints are previously described. These include acute inflammation, mural necrosis and vascular thrombosis in early stages (48 hours), fibrosis with foreign body type giant cells and mononuclear inflammatory cells in intermediate stages (1 month), and foreign body granulomas surrounding cyanoacrylate in vessels and parenchyma (6 months) [3].

We report foreign body type giant cell reaction, inflammation, ulceration, and mass formation in the stomach caused by cyanoacrylate injections, in the context of endoscopic biopsies and gastric resections. Pathologists and clinicians should be aware of such long-term complications of “glue” injection into varices or bleeding sites and realise that this material can also form a discreet mass in the stomach wall.

## 2. Case  1

**Figure 1:**
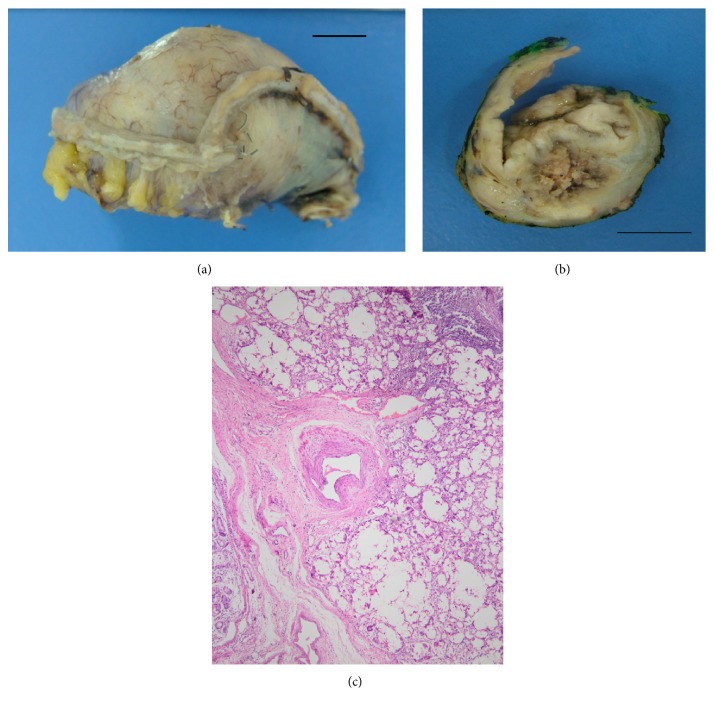
*Case  1*. The gastric resection specimen's (a) cut surface (b) revealed a grey to tan colored, friable lesion with a necrotic center. Scale bar = 1 cm. A submucosal, multinodular, “bubbly” lesion with accompanying chronic inflammatory infiltrate was noted ((c) ×10, hematoxylin-eosin).

A 61-year-old male underwent distal pancreatectomy and splenectomy in September 2013, after 11 rounds of chemotherapy for metastatic pancreatic adenocarcinoma. A mass was detected in the stomach wall intraoperatively. This mass was considered to be a metastasis of pancreatic adenocarcinoma and was resected. The lesion had caused irregularity at the serosal surface of the stomach and was hard in consistency. The cut section of this 5 × 3 × 1.5 cm-sized gastric resection specimen revealed a poorly circumscribed white lesion, 1.5 cm in diameter, that spanned the whole gastric wall except for the mucosa, with grey-white necrotic material in its center (Figures 1(a) and 1(b)).

**Figure 2:**
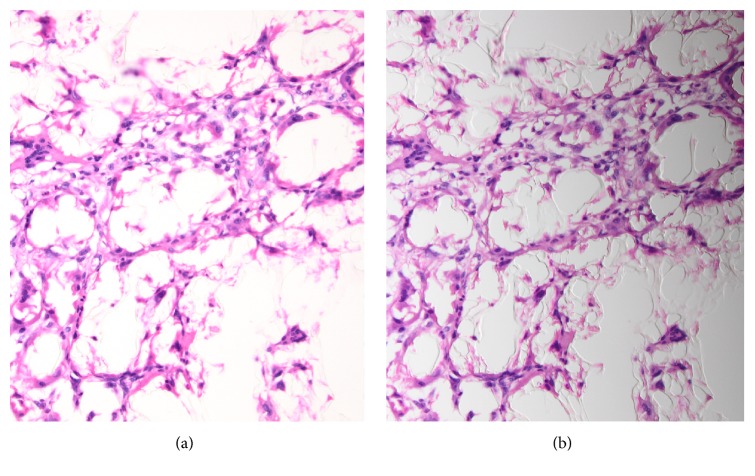
The lesion in Case 1 consisted of multinucleated giant cells surrounding clear appearing cystic spaces ((a) ×20). Slight dimming of the light source on one side revealed translucent material rimming these spaces ((b) ×20).

A representative section revealed a bubbly, cystic, coarsely nodular lesion with occasional necrotic foci. Numerous foreign body type multinucleated giant cells surrounded empty-looking spaces. The outer margins of the lesion harbored a mild to moderate lymphoplasmacytic infiltrate. There were scattered hemosiderin-laden macrophages that suggested prior erythrocyte extravasation. The mucosa was intact; the lesion spanned the submucosa and destroyed the muscularis propria, reaching the serosa (Figure 1(c)). Closer inspection revealed translucent, wispy material that rimmed the spaces giant cells had encircled (Figure 2(a)). This material did not fully refract polarized light, but slight dimming of the light source made the material more visible, rendering the material “slightly refractile” (Figure 2(b)). Patient's history revealed a bout of gastric bleeding and transfusions 2 months before the resection. The hemorrhage was then treated by cyanoacrylate injection.

## 3. Case  2

A 52-year-old female with a history of infertility was admitted with abdominal pain, nausea, and vomiting in December 2007. A hepatobiliary ultrasonography revealed portal vein thrombosis. Abdominal CT scan showed thrombosis in superior mesenteric and renal veins. Anticoagulant therapy was started. In August 2008, gastroesophageal varices were detected in upper gastrointestinal endoscopy. In July 2009, she presented with upper gastrointestinal bleeding. Endoscopy revealed varices in the distal esophagus and fundus of the stomach, with an overlying clot. 0,5 mL of glue was injected to esophageal varices. Afterwards the patient underwent gastrostomy and ligation. In August 2010 gastric variceal bleeding recurred and was embolised with 3% beta-blocker, alcohol, and 30% glue injection. In 2011, 2012, and 2013, follow-up endoscopies persistently displayed gastric varices and histoacryl glue injections were administered to ulcerated gastroesophageal varices.

**Figure 3:**
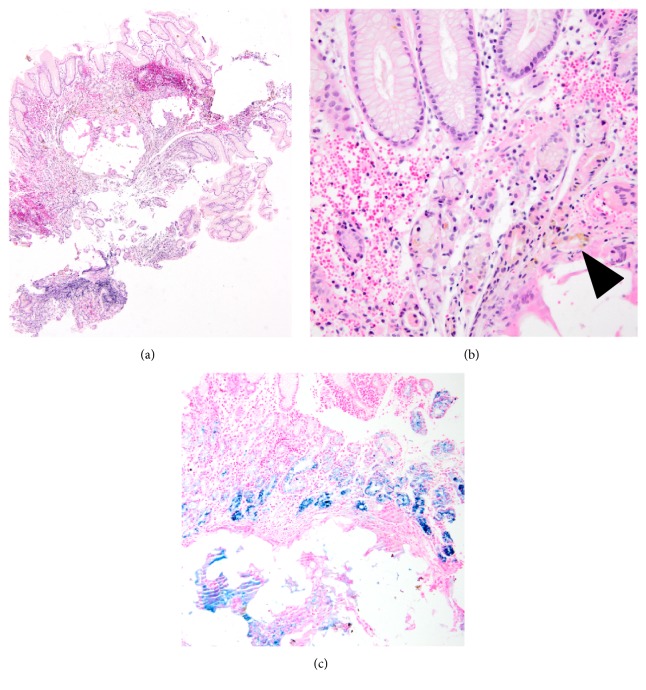
Case 2 consisted of a hemorrhagic mucosal tissue with multiple small spaces and chronic inflammation in lamina propria ((a) ×2). Mucosal glands were disrupted by nodules of foreign body type giant cells surrounding vaguely globoid spaces; hemosiderin (brown pigment, arrowhead) was visible in nearby cells ((b) ×40). Gastric glands and giant cells displayed siderosis ((c) Prussian Blue, ×10).

In 2015, a biopsy was taken from an ulcerated lesion in fundic and antral mucosa. On histopathologic examination, focal ulceration and exudate were seen in the mucosa. Beneath the intact part of the epithelium, there were extravasated erythrocytes (Figure 3(a)). In the gastric wall, translucent spaces and giant cell reaction around these spaces were present. Lymphocytic aggregates and lymphoplasmacytic inflammatory reaction were also seen (Figure 3(b)). Prussian Blue staining displayed siderosis in giant cells and gastric glands (Figure 3(c)).

## 4. Case  3

A 56-year-old female with chronic viral B hepatitis and cirrhosis was admitted in December 2011. Glue injection was performed to treat gastric variceal bleeding. In July 2012, the patient had an upper gastrointestinal endoscopic examination. An ulcerated lesion, 2 cm in diameter, was seen in the fundic mucosa and the edge of the ulcer was biopsied.

**Figure 4:**
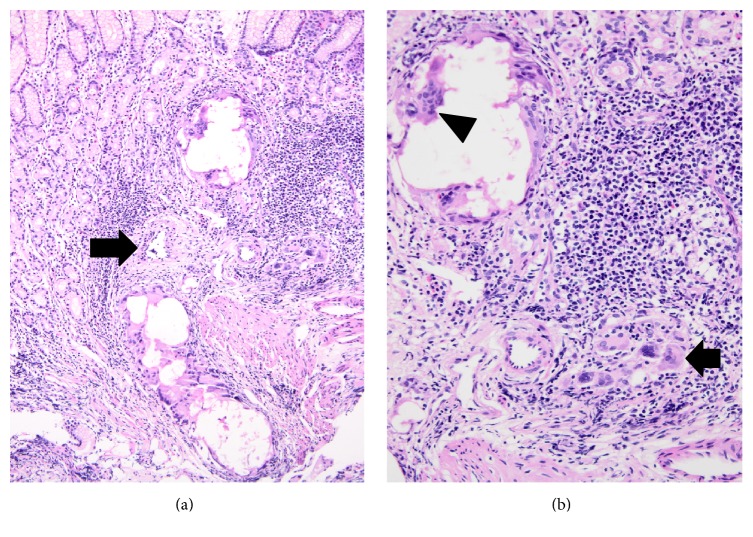
Case 3 demonstrated a trace of a vessel (arrow) that was engorged with cyanoacrylate, here seen as a trace of cystic-globoid structures ((a) ×10). Closer view of these structures showed multinucleated giant cells around the spaces (arrowhead) and chronic inflammatory reaction (arrow) in lamina propria ((b) ×20).

The foveolar epithelium was intact, but beneath the epithelium there were extravasated erythrocytes and edema. A trace of foreign body giant cell reaction was detected in the lamina propria. Translucent foreign material was present in the cytoplasm of giant cells. An inflammatory reaction composed of eosinophils and lymphoid aggregates was detected (Figures 4(a) and 4(b)). The foreign material had settled into the lamina propria in a curvilinear fashion, which could suggest a vascular trace. The paucity of the remaining tissue precluded additional studies to prove the presence of an endothelial lining, but the pathogenesis and the curvilinear orientation of the lesion both support the presence of an involved vessel in this particular case (Figure 4(a)).

## 5. Discussion

Granulomatous gastritis can appear in a number of clinical settings: Crohn's disease, sarcoidosis, infections, lymphoma, adenocarcinoma, Whipple's disease, Langerhans cell histiocytosis, gastric perforation, and vasculitis [1]. The concept of idiopathic granulomatous gastritis is under debate [1]. Here, we report the presence of foreign body type granulomas, chronic inflammation in gastric endoscopic biopsies, and resections as a complication of cyanoacrylate injection.

Cyanoacrylate is a well-known tissue adhesive used in the treatment of varices related to portal hypertension, vascular malformations, and bleeding episodes of these lesions [5]. It typically polymerizes in ionic environment. The rate of polymerization can be adjusted with additional nonionic agents depending on the blood flow properties of the lesion at hand [3]. The polymerization process creates heat, which causes the release of acrylacetate and formaldehyde [5]. Common complications of cyanoacrylate treatment include ulceration and rebleeding. Fever and emboli to various organs can also occur, along with abscess and fistula formation [6]. Abscesses are reported in association with the stomach [6], brain [7], and peritoneal cavity [8]. An animal study using N-butyl-2-cyanoacrylate reported acute inflammation in the injection site in the first week and granulomatous vasculitis and fibrosis appearing after 2 months [5].

In these three cases described above, none of them were diagnosed with a granuloma-causing disease during follow-up. Special stains for microorganisms were negative in all cases. There were no evidence for gastric pneumatosis in abdominal CTs. All cases had a history of glue injection to treat the bleeding. The gastric biopsies were sampled 2 months (Case 1), 7 months (Case 3), or 6 years (Case 2) after the initial vascular embolisation. All cases displayed a similar “bubbly” appearance with “slightly refractile” translucent material and surrounding chronic inflammation with the tissue findings of old and new hemorrhage.

Cyanoacrylate-related findings in gastric biopsies have not been reported previously. Cyanoacrylate has the ability to harden and induce intense inflammatory reaction and fibrosis in and around the injection sites. These reactive tissue changes can be mistaken for a neoplastic mass, depending on the clinical context. The main morphological finding is to see “slightly refractile” wisps of foreign material in the empty-looking spaces with accompanying foreign body type giant cells. Ulceration and bleeding are also seen around the foreign material. The history of previous cyanoacrylate embolisation for variceal or gastric bleeding is critical for diagnosis. In association with the histopathological changes as described above, positive history for glue injection and absence of specific etiology to explain granulomatous gastritis are diagnostic for cyanoacrylate associated granulomatous gastritis.
